# Biallelic novel mutations of the COL27A1 gene in a patient with Steel syndrome

**DOI:** 10.1038/s41439-021-00149-7

**Published:** 2021-05-07

**Authors:** Jong Seop Kim, Hyoungseok Jeon, Hyeran Lee, Jung Min Ko, Yonghwan Kim, Murim Choi, Gen Nishimura, Ok-Hwa Kim, Tae-Joon Cho

**Affiliations:** 1 Division of Pediatric Orthopaedics, Seoul National University Children’s Hospital and Seoul National University College of Medicine, Seoul, Republic of Korea; 2 Department of Biomedical Sciences, Seoul National University College of Medicine, Seoul, Republic of Korea; 3 Department of Pediatrics, Seoul National University Children’s Hospital and Seoul National University College of Medicine, Seoul, Republic of Korea; 4 Department of Biological Sciences, Sookmyung Women’s University, Seoul, Republic of Korea; 5 Center for Intractable Disease, Saitama Medical University Hospital, Saitama, Japan; 6 Department of Pediatric Radiology, VIC365 Children’s Hospital, Incheon, Republic of Korea

**Keywords:** Genetics research, Medical genetics

## Abstract

An 11-year-old Korean boy presented with short stature, hip dysplasia, radial head dislocation, carpal coalition, genu valgum, and fixed patellar dislocation and was clinically diagnosed with Steel syndrome. Scrutinizing the trio whole-exome sequencing data revealed novel compound heterozygous mutations of *COL27A1* (c.[4229_4233dup]; [3718_5436del], p.[Gly1412Argfs*157];[Gly1240_Lys1812del]) in the proband, which were inherited from heterozygous parents. The maternal mutation was a large deletion encompassing exons 38–60, which was challenging to detect.

Steel syndrome, which is characterized by short stature, hip dislocation, radial head dislocation, and carpal coalition, was first described in 23 Puerto Rican children^1^. It is inherited as an autosomal recessive trait^1,2^, and biallelic loss-of-function mutations of the *COL27A1* gene were found to be pathogenic for this phenotype^3^. Only a few cases were reported who are not Puerto Rican descendants^4–10^.

An 11-year-old boy presented with right knee discomfort and limping. He was the only child of a healthy nonconsanguineous Korean couple. His birth weight was 2.5 kg (*z* = −2), and routine newborn examinations revealed bilateral cryptorchidism and bilateral hearing impairment. At 1 year of age, he underwent orchiopexy and was provided with a hearing aid. Motor and cognitive development has been typical. A retrospective review of radiographs at age 5 revealed a vertebral anomaly (mild thoracolumbar kyphoscoliosis with a hypoplastic vertebral body of L1), hip dysplasia, and genu valgum, as well as an unusually large capitate and small hamate. The epiphyses of the knee were unusually round (Figs. 1 and 2). At 11 years of age, his height was 120.5 cm (*z* = −3.2), and his arm span was 107 cm. Physical examination revealed prominent forehead, hypertelorism, broad nasal bridge, midface hypoplasia with mild prognathism, pectus excavatum, short hands, genu valgum, and fixed patellar dislocation on the right side, flexion contracture of the left elbow, and marked out-toeing gait on both sides. A repeat skeletal survey at 11 years of age recapitulated what was previously seen, plus progressive kyphoscoliosis, multiple sites of the carpal coalition, and fixed patellar dislocation. These findings were compatible with Steel syndrome. Femoral retroversion and external tibial torsion were confirmed by computed tomography of the lower extremities. The hip was dysplastic but not dislocated (Figs. 1 and 2).

**Fig. 1:**
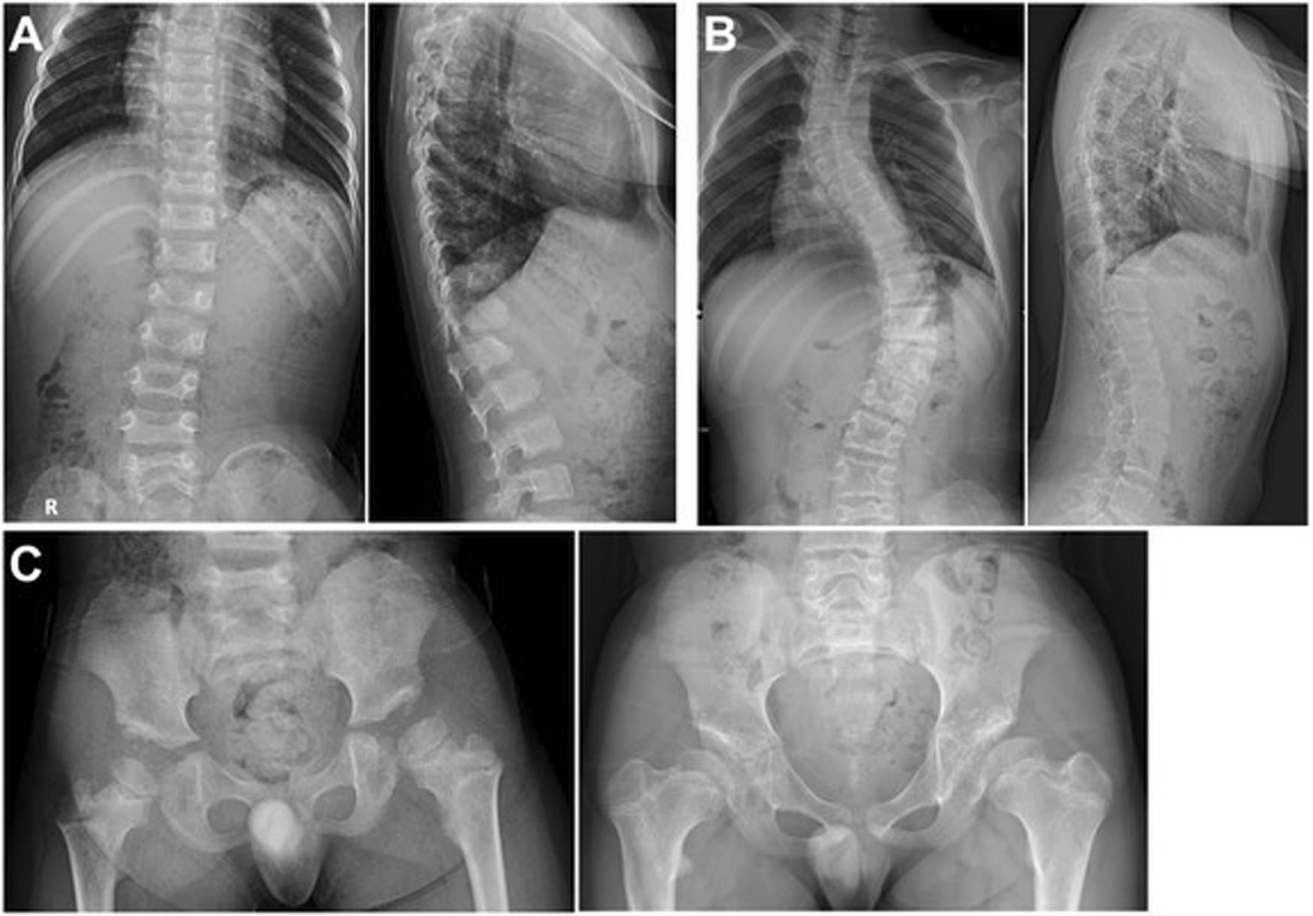
Radiographs of the spine and hip. **A** Radiographs of the spine at 5 years of age showing mild scoliosis and L1 dysplasia with anteroinferior beaking. **B** Radiographs of the spine at 11 years of age showing progressed thoracolumbar scoliosis. **C** Hip radiograph at 5 years of age (left) showing hip dysplasia with a flat and irregular acetabular roof and at 11 years (right) showing a steep acetabular roof. The proximal femur had coxa breva and coxa vara deformities. The femoral head was poorly covered by the acetabulum but not dislocated.

**Fig. 2:**
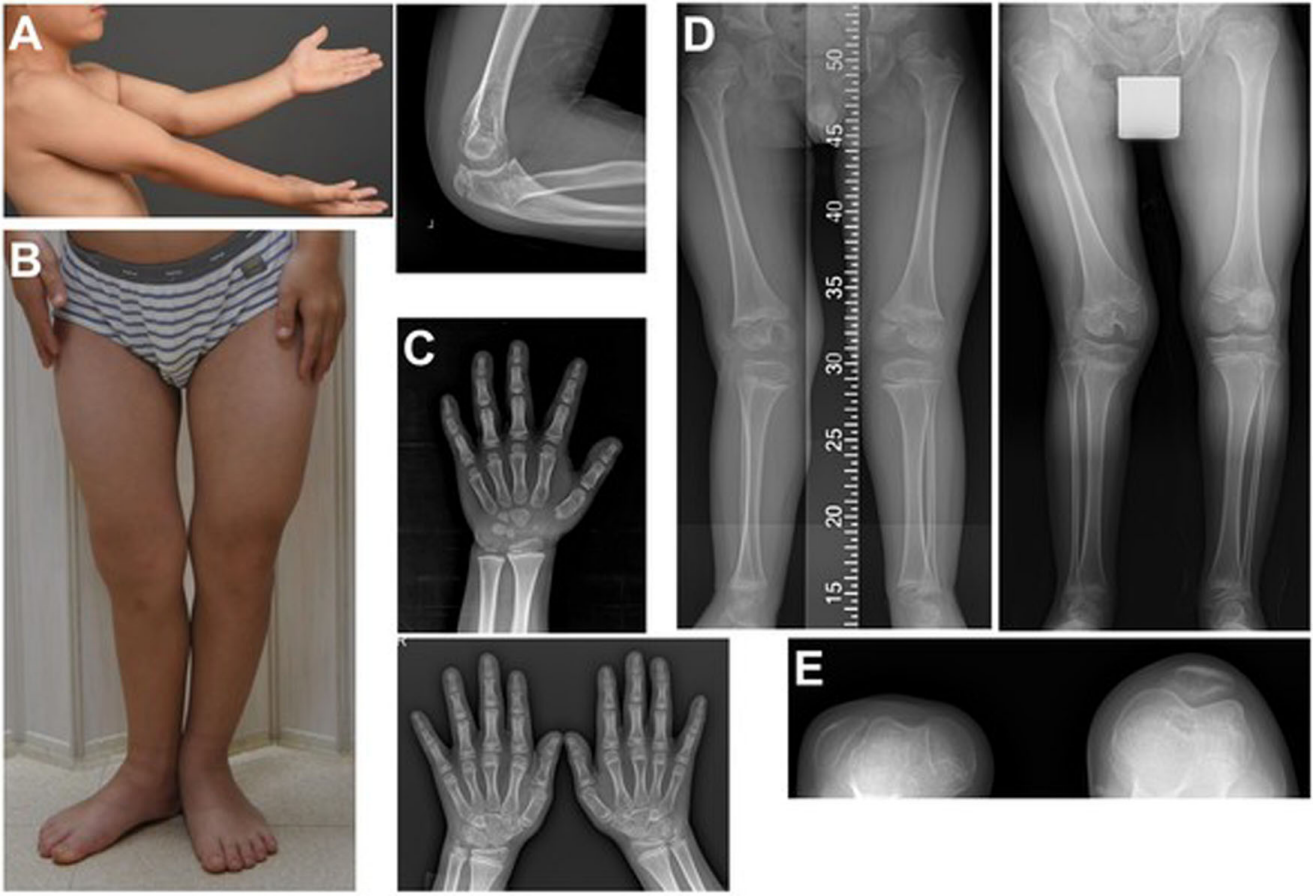
Photographs and radiographs of the extremities. **A** A photo taken at 11 years of age showing elbow flexion contracture and a radiograph showing radial head dislocation on the left side. **B** A photo taken at 11 years of age showing externally rotated lower extremities. The right genu valgum deformity was not visualized well due to an externally rotated position. **C**. A radiograph of the hand at 5 years of age (upper) barely showing any finding of the carpal coalition, and one at 11 years (lower) showing evident bilateral sites of carpal coalition. **D**, **E** Radiographs at 5 and 11 years of age showing aggravating genu valgum with the asymmetrical shape of the right distal femoral epiphysis and patellar dislocation.

To identify causal variants in the proband, we performed whole-exome sequencing on the proband and parents using genomic DNA extracted from the circulating leukocytes following a previous study^11^. We paid special attention to *COL27A1* because the proband phenotype was compatible with Steel syndrome. Initially, we found a homozygous frameshift 5-nucleotide insertion variant of *COL27A1* (NM_032888.4: c.4229_4233dup, p.Gly1412Argfs*157) in the proband, the father was heterozygous for this variant, and this variant was absent in the mother (Fig. 3A and Supplementary Table S1). Then, we checked the copy number status of *COL27A1* by comparing the coverage depth of each family member and found that the father’s coverage of exons 38–60 was approximately twice as high as that of the mother and proband (Fig. 3B). RT-PCR of mRNA extracted from the proband’s dermal fibroblast using primers at exons 37 and 61 (Supplementary Table S1) confirmed heterozygous deletion of these exons (c.3718_5436del, p.Gly1240_Lys1812del) (Fig. 3C). This large deletion encompassed the 5-nucleotide insertion variant, explaining why the insertion variant in the proband appeared to be homozygous. These two variants have not been recorded in the Genome Aggregation Database (gnomAD; https://gnomad.broadinstitute.org/) and in-house database of ~5000 Korean individuals without obvious skeletal dysplasia. Overall, we concluded that the proband inherited the heterozygous deletion of 23 exons from the mother, which is expected to confer a substantial alteration in protein function, and the heterozygous 5-nucleotide insertion variant from the father, which would cause a null effect (Fig. 3D).

**Fig. 3:**
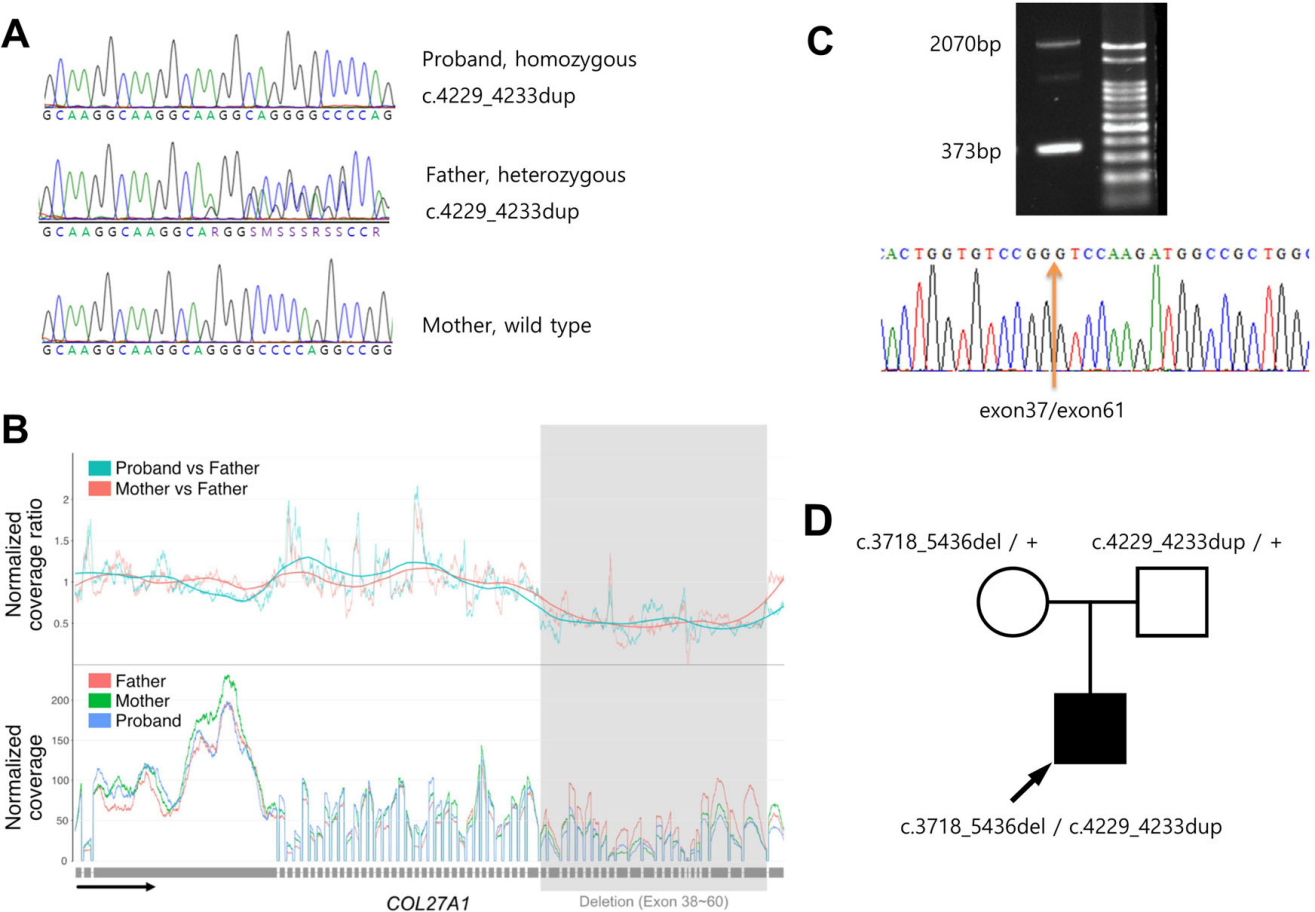
Results of genetic testing. **A** Sanger sequencing showing the c.4233_4234insAGGCA variant, which is homozygous in the proband and heterozygous in the father. **B** Normalized coverage of NGS showing deletion of exons 38–60 in the proband and mother. **C** RT-PCT using primers at exons 37 and 61 and Sanger sequencing of the amplicon showing the presence of transcripts without exons 38–60 (373â€‰bp) along with a wild-type transcript (2070â€‰bp). **D** Pedigree of the family with *COL27A1* genotype.

The present boy had cryptorchism and hearing impairment, from which it was ascertained that some extraskeletal features are syndromic components of Steel syndrome, as previously reported^4,6–8,10^. As described in previous reports, his skeletal abnormalities are major clinical concerns. He showed kyphoscoliosis with L1 hypoplasia, radial head dislocation, acetabular and proximal femoral dysplasia, genu valgum, patellar dislocation, external torsional deformity of the lower extremities with marked out-toeing, and multiple sites of the carpal coalition. These findings have more or less been described in previous reports^1–5,8,9,12^. Although hip dislocation has been reported in most previous reports^6–10,12,13^, the present boy had acetabular and proximal femoral dysplasia but no dislocation, as found in some individuals previously^4,5^. Hence, hip pathology in Steel syndrome should range from acetabular dysplasia to complete dislocation. He underwent a series of surgical interventions to address the torsional deformity of the lower extremities, hip dysplasia, genu valgum, and patellar dislocation. From a radiological viewpoint, the hypoplasia of L1 and round epiphyses of the knee deserve comments. The former might lead to a misdiagnosis of spondyloepiphyseal dysplasia, while the latter might lead to a misdiagnosis of other joint laxity syndromes, such as Larsen dysplasia, unless carpal coalition is noted.

Nineteen individuals with molecularly confirmed Steel syndrome have been reported so far. Eleven of them were Puerto Rican descendants and shared the same founder mutation: homozygous c.2089G>C, p.Gly697Arg^3,12,13^. Individuals with Steel syndrome who are not Puerto Rican descendants have either compound heterozygous mutations^8–10^ or homozygous mutations of *COL27A1* that are different from the Puerto Rican founder mutation^4–7^. The mutations identified in this report are unique in that one is a 5-nucleotide insertion and the other is a large deletion encompassing 23 exons. Both of them are predicted to cause a substantial reduction in the function of COL27A1. Because the phenotype description is variable among the reports, it was almost impossible to conduct a genotype–phenotype correlation. A reinterpretation and summary of previously reported mutation-confirmed individuals with Steel syndrome are listed in Supplementary Table S2.

For the individual in this report, it was challenging to detect the large deletion, which was finally detected by scrutinizing the exome data based upon reassurance of the Steel syndrome phenotype. This case could serve as an example showing how important it is to correlate the next-generation sequencing data with the phenotype.

## Supplementary information

Table S1

Table S2

## Data Availability

The relevant data from this Data Report are hosted at the Human Genome Variation Database at 10.6084/m9.figshare.hgv.3002; 10.6084/m9.figshare.hgv.3005.
