# Supplementary material for: Biallelic novel mutations of the COL27A1 gene in a patient with Steel syndrome
Source: Hum Genome Var. 2021 May 7;8:17. doi: 10.1038/s41439-021-00149-7 (PMC8105406; doi:10.1038/s41439-021-00149-7)
Supplement: Supplementary file 1 — Table S1 [file 41439_2021_149_MOESM1_ESM.docx]

**Table S1.** Sequence of primers used in this study

| Primer | Sequence | Product size | Location |
| --- | --- | --- | --- |
| Sanger_F^1^ | 5'-GGCCCACAATCCTCTCAG-3' | 486bp | Exon 46 |
| Sanger_R^1^ | 5'-ATGTGTTGGTTCTGCCCATC-3' |  | Exon 46 |
| RTPCR_F^2^ | 5'-GATGAAGGGTGACCTTGGAC-3' | 2070bp^3^ / 373bp^4^ | Exon 37 |
| RTPCR_R^2^ | 5'-CCAACTTCCAGGCGGTACT-3' |  | Exon 61 |

^1^ Sanger sequencing for c.4233_4234insAGGCA variant; ^2^ RT-PCR for c.3718_5436del variant;

^3^ Predicted product size in wild type; ^4^ Predicted product size in variant
