# Supplementary material for: Biallelic novel mutations of the COL27A1 gene in a patient with Steel syndrome
Source: Hum Genome Var. 2021 May 7;8:17. doi: 10.1038/s41439-021-00149-7 (PMC8105406; doi:10.1038/s41439-021-00149-7)
Supplement: Supplementary file 2 — Table S2 [file 41439_2021_149_MOESM2_ESM.docx]

| **Table S2. Summary of the genotype and phenotype of the individuals with mutation-confirmed Steel syndrome** | | | | | | | | | | | | | | | | |  |
| --- | --- | --- | --- | --- | --- | --- | --- | --- | --- | --- | --- | --- | --- | --- | --- | --- | --- |
|  | **Ref** | **Genotype** | **Ethnicity** | **Gender** | **Age at diagnosis** | **Stature; body proportion** | **Development** | **Face** | **Trunk and spine** | **Hip** | **Lower limb** | **Foot** | **Upper limb and elbow** | **Carpal coalition** | **Hand** | **Other manifestations** | |
| 1 | Ref 3 | c.2089G>C, homo | Puerto Rican | M | 13yr | <5% | normal intelligence | facial dysmorphism, mild | scoliosis; odontoid hypoplasia | bilat dislocation; poorly ossified femoral head | n.a. | bilat pes planus | unilat RH dislocation | bilat capitate-hamate | bilat 5th finger clinodactyly |  | |
| 2 | Ref 3 | c.2089G>C, homo | Puerto Rican | F | 22mo | <5% | normal intelligence | facial dysmorphism | lordosis; scoliosis | bilat dislocation; poorly ossified femoral head | n.a. | n.a. | none | bilat capitate-hamate | bilat 5th finger clinodactyly |  | |
| 3 | Ref 3 | c.2089G>C, homo | Puerto Rican | F | at birth | n.a. | n.a. | n.a. | n.a. | bilat dislocation | n.a. | n.a. | n.a. | n.a. | n.a. |  | |
| 4-8 [1] | Ref 13 | c.2089G>C, homo | Puerto Rican | 3F 2M | mean 51.6yr | <-2SD | n.a. | n.a. | 2 scoliosis; 3 cervical stenosis; 1 cervical discitis; 2 cervical spondylosis; 3 cervical cord compression | 4 dislocation; 1 LLD | n.a. | n.a. | 1 contracture; 1 wrist deformity; 2 lunotriquetral fusion | n.a. | n.a. |  | |
| 9 | Ref 4 | c.3556-2A>G, homo | Emirati | F | 3yr | N.I. | normal motor; delayed speech | facial dysmorphism | C3-4 fusion, C4-5 increased disk height | bilat dysplasia [3] | genu valgum | n.a. | radial head dislocation [3] | n.a. | n.a. | bilat SN hearing loss | |
| 10 | Ref 10 | c.521_528del; c.2119C>T | Indian | F | 3yr | -3SD | delayed motor (walk alone 2yr) | facial dysmorphism | n.a. | bilat dislocation | genu valgum | bilat foot deformity [3] | none [3] | capitate-hamate | syndactyly of fingers and toes | hydronephrosis | |
| 11 | Ref 9 | c.93del; c.3075del | Syrian | F | 9yr | -7.9SD | motor delay | facial dysmorphism | severe kyphoscoliosis | bilat dislocation | marked external torsion | vertical talus | n.a. | n.a. | n.a. | SN hearing impairment; coloboma | |
| 12 | Ref 6 | c.2710G>A, homo | Iraqi | F | n.a. | n.a. | intellectual disability [2] | hypertelorism | wide spaced nipples | bilat dislocation | n.a. | pes cavus | short upper limbs | n.a. | n.a. | hearing impairment | |
| 13 | Ref 7 | c.4261-1G>A, homo | Yemeni | M | 5yr | n.a. | delayed | facial dysmorphism | scoliosis | dislocation | n.a. | bilat foot deformity [3] | n.a. | n.a. | n.a. | cryptorchidism, inguinal hernia | |
| 14 | Ref 12 | c.2089G>C, homo | Puerto Rican | M | 7yr | <5% | no delay | facial dysmorphism | n.a. | bilat dislocation | n.a. | n.a. | bilat RH dislocation | n.a. | n.a. |  | |
| 15 | Ref 12 | c.2089G>C, homo | Puerto Rican | F | 2mo | 5~10% | no delay | facial dysmorphism | pectus excavatum; mild scoliosis | hip dysplasia [4] | out-toeing | n.a. | n.a. | n.a. | n.a. | laryngomalacia; oromotor dysphagia needing G-tube; mild hypotonia and ligament laxity | |
| 16 | Ref 12 | c.2089G>C, homo | Puerto Rican | M | 14yr | 50% | gross motor delay | facial dysmorphism | scoliosis | bilat dislocation | n.a. | metatarsus adductus; talocalcaneal coalition | bilateral RH dislocation | n.a. | n.a. |  | |
| 17 | Ref 5 | c.2405G>A, homo | Greek-Cypriot | M | 4yr | -2SD; mesomelic | delayed speech | facial dysmorphism; open fontanelle at 11mo | pectus excavatum; anterior notching of upper lumbar vertebra | no dislocation; delayed femoral head ossification at 11mo | genu valgum, out-toeing | n.a. | n.a. | n.a. | 2nd-3rd incomplete syndactyly | bilat. SN hearing loss at 3+10yr | |
| 18 | Ref 8 | c.2026G>C; c.2367G>A | Japanese | M | 5yr | -1.26SD | social and language delay | facial dysmorphism | none | bilat dislocation | bilat genu valgum and patellar dislocation | right congenital vertical talus, left talipes valgus | RH dislocation, 5th finger clinodactyly, syndactyly | n.a. | n.a. | SN hearing impairment, atrial septal defect, small penis, umbilical hernia | |
| 19 | Ref 8 | c.2026G>C; c.2367G>A | Japanese | M | 3yr | -0.97SD | delayed | facial dysmorphism | none | left dislocation | left patellar dislocation | bilat talipes valgus | RH dislocation, 5th finger clinodactyly, syndactyly | n.a. | n.a. | hearing , small penis, cryptorchidism | |
| 20 | Present study | c.4229_4233dup; c.3718_5436del | Korean | M | 11yr | -3.2SD | no delay | facial dysmorphism | scoliosis | bilat acetabular dysplasia | Rt. genu valgum and patellar dislocation | n.a. | unilat RH dislocation | bilat multifocal | n.a. | hearing impairment, cryptorchidism | |
| n.a.: not available or not described ; bilat: bilateral ; unilat: unilateral ; RH: radial head ; LLD: leg length discrepancy ; SN: sensorineural | | | | | | | | | | | | | | | | |  |
| [1] Five individuals were described as a group. [2] The authors suggested that FRMD4A VUS may contribute to this phenotype. [3] Re-interpreted based on the displayed figure. [4] It is not clear whether to imply dislocation or acetabular dysplasia. [5] It seems to imply radial head dislocation. | | | | | | | | | | | | | | | | |  |
